# Diagnostic Yield of Transabdominal Ultrasonography for Evaluation of Pancreatic Cystic Lesions Compared with Endoscopic Ultrasonography

**DOI:** 10.3390/jcm10194616

**Published:** 2021-10-08

**Authors:** Yu Ji Li, Gil Ho Lee, Min Jae Yang, Jae Chul Hwang, Byung Moo Yoo, Soon Sun Kim, Sun Gyo Lim, Jin Hong Kim

**Affiliations:** Department of Gastroenterology, Ajou University School of Medicine, 164 Worldcup-ro, Yeongtong-gu, Suwon-si 443-380, Korea; mikou2000@163.com (Y.J.L.); micorie@hanmail.net (G.H.L.); cath07@naver.com (J.C.H.); ybm6403@gmail.com (B.M.Y.); cocorico99@gmail.com (S.S.K.); mdlsk75@ajou.ac.kr (S.G.L.)

**Keywords:** endosonography, pancreatic cyst, pancreatic intraductal neoplasms, pancreatic neoplasms, ultrasonography

## Abstract

Detection rates of pancreatic cystic lesions (PCLs) have increased, resulting in greater requirements for regular monitoring using imaging modalities. We aimed to evaluate the capability of ultrasonography (US) for morphological characterization of PCLs as a reference standard using endoscopic ultrasonography (EUS). A retrospective analysis was conducted of 102 PCLs from 92 patients who underwent US immediately prior to EUS between January 2014 and May 2017. The intermodality reliability and agreement of the PCL morphologic findings of the two techniques were analyzed and compared using the intraclass correlation coefficient and κ values. The success rates of US for delineating PCLs in the head, body, and tail of the pancreas were 77.8%, 91.8%, and 70.6%, respectively. The intraclass correlation coefficient for US and the corresponding EUS lesion size showed very good reliability (0.978; *p* < 0.001). The κ value between modalities was 0.882 for pancreatic duct dilation, indicating good agreement. The κ values for solid components and cystic wall and septal thickening were 0.481 and 0.395, respectively, indicating moderate agreement. US may be useful for monitoring PCL growth and changes in pancreatic duct dilation, but it has limited use in the diagnosis and surveillance of mural nodules or cystic wall thickness changes.

## 1. Introduction

Pancreatic cystic lesions (PCLs) are a group of diseases characterized by different pathological processes, ranging from benign to malignant [1]. Due to improvements in cross-sectional imaging technology and the increase in the frequency of regular health check-ups, PCLs are becoming more easily detected. The incidence of PCLs worldwide varies greatly from 0.2% to 49.1% [2]. Korea is reported to have an incidence of 0.47%, of which 34% of patients are asymptomatic [3]. The incidence of a PCL developing into precancerous or malignant lesions has reached 47% [4]. Intraductal papillary mucinous neoplasms and mucinous cystic neoplasms are common PCLs with a potential or obvious malignant tendency. It has been reported that low-risk intraductal papillary mucinous neoplasms, even without any worrisome features, have a 0.6% to 0.8% annual chance of developing into cancer within 10 years [5].

At present, the representative diagnostic methods for PCL are computed tomography (CT) imaging, magnetic resonance imaging (MRI), and endoscopic ultrasonography (EUS). Although CT is the most common imaging method, there is a risk of radiation- or contrast-induced injury to patients, and its diagnostic reliability is inferior to that of MRI or EUS. In contrast, MRI uses no radiation and produces a high-resolution image of soft tissue, but it is expensive and time-consuming. EUS is an imaging modality that can facilitate imaging in closer proximity to the pancreas, and it has a high spatial resolution; however, it is relatively invasive and highly operator-dependent [6].

Although abdominal ultrasonography (US) is not currently a standard imaging modality for the visualization and diagnosis of PCLs, it has the advantages of being cost-effective, not emitting radiation, and being a simple, non-invasive examination procedure [7]. Until now, there has been little published literature regarding the role of US in the diagnosis and follow-up of PCLs. Therefore, in the current study, we aimed to evaluate the capability of US for the morphological characterization of PCLs as a reference standard using EUS.

## 2. Materials and Methods

### 2.1. Patients

Patients with PCLs who were examined between January 2014 and May 2017 were retrospectively enrolled in this study. These patients underwent US examination prior to EUS on the same day. The exclusion criteria were as follows: (1) previous history of pancreatic cyst aspiration; (2) a lesion that was presumed to be a cyst on US, but eventually turned out to be a pure solid lesion on EUS; and (3) a PCL that could not be delineated using EUS. This study was approved by the Institutional Review Board of Ajou University Hospital (Approval number: AJIRB-MED-MDB-21-034), and informed consent was obtained from each patient undergoing US and EUS.

### 2.2. Image Evaluation

US and EUS were performed by one physician with 35 years of clinical experience in conducting US examinations and 20 years of experience in EUS. First, the pancreas was carefully investigated using a convex US transducer with a frequency of 3.5 MHz (Aplio500; Canon Medical Systems, Otawara, Japan). For detailed evaluation of the pancreas, the US included transverse and oblique scan planes at different levels. The spleen was often used as a sonic window to visualize the tail of the pancreas. EUS was subsequently performed using a radial/linear echoendoscope (GF-UE260-AL5/GF-UCT260; Olympus Corp., Tokyo, Japan) under moderate sedation. EUS was conducted in various planes, with both transgastric and tranduodenal access to scan the whole pancreas, detect the PCL, and characterize its morphology. Imaging analysis was performed using INFINITT PACS 3.0.11.3 BN104 (INFINITT Healthcare Co., Seoul, Korea).

### 2.3. Outcome Measures

A cyst was defined as an anechoic lesion with posterior enhancement on US or EUS images. The locations of individual PCLs were categorized into three groups based on which of the following sections of the pancreas they presented on: the head, body, or tail. The size of the PCL was defined as the longest dimension measured via US or EUS. Delineation success was defined as the successful detection of a PCL with US at the same location as that of the EUS reference image. The morphological characteristics of each PCL were described according to the locularity (unilocular, oligolocular, or multilocular), outer margin appearance (smooth, lobulated, or irregular), size of individual compartments of cysts (microcystic, macrocystic, or mixed), shape of individual compartments of cysts (pleomorphic, grape-like, sponge-like, or finger-like), presence of solid components, and main pancreatic duct dilation [8,9,10]. For quality control, all the original US and EUS images were reevaluated by an independent investigator who was blinded to the results of the other modalities.

### 2.4. Statistical Analyses

The intermodality reliability and the agreement of the morphologic findings of PCLs between the US and EUS modalities were analyzed and compared. The PCL sizes and locations derived from the US images were compared with the corresponding EUS findings using intraclass correlation coefficients (ICC) applied 2-way mixed model and type of consistency. An ICC is usually used to assess a technique’s reliability, which refers to the consistency of repeatedly measured values for the same object. According to the value of the ICC, the reliability between different methods is categorized as follows: an ICC value of >0.80, very good; ICC of 0.60–0.79, moderate; ICC < 0.60, not reliable [11]. The agreement for detailed morphologic characteristics of PCLs, including worrisome features, between US and EUS was evaluated using the κ value. The κ value (strength of agreement) is categorized as follows: κ of 0.81–1.00, excellent; κ of 0.61–0.81, good; κ of 0.41–0.60, moderate; κ of 0.21–0.40, fair; and κ of 0–0.20, poor [12]. Statistical analysis was performed using IBM SPSS Software version 20.0 for Windows (IBM Corp., Armonk, NY, USA).

## 3. Results

**Figure 1:**
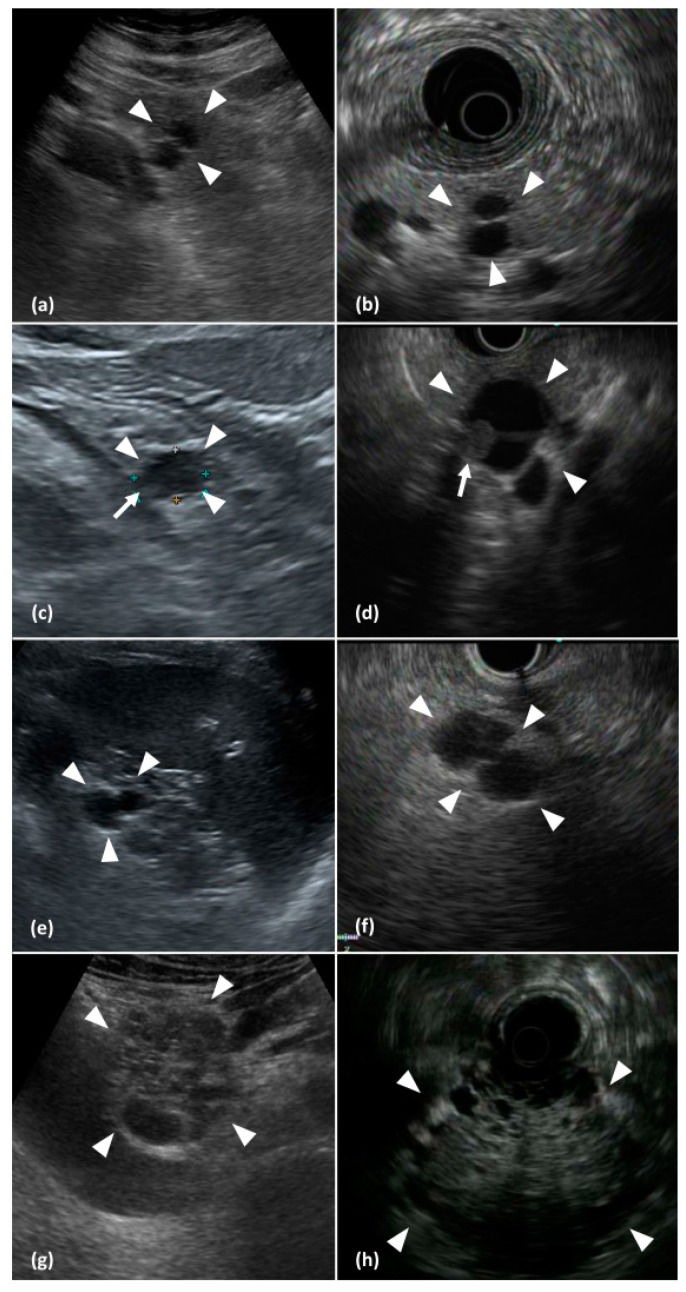
Pancreatic cystic lesions detected on abdominal ultrasonography (US; left column images) and corresponding endoscopic ultrasonography (EUS; right column images). (**a**,**b**) A bilocular cystic lesion (arrowheads) with a thin internal septum in the body of the pancreas. (**c**,**d**) An oligolocular cystic lesion (arrowheads) with a solid component (arrows) in the tail of the pancreas. (**e**) An oligolocular cystic lesion (arrowheads) detected on a transsplenic scan of the pancreatic tail and the corresponding EUS image (**f**). (**g**,**h**) A large microcystic lesion (arrowheads) with a honeycomb appearance in the head of the pancreas.

A total of 102 PCLs from 92 patients were included in this study. Patient demographics and PCL characteristics are summarized in Table 1. Some representative images of PCLs are presented in Figure 1. The PCLs were located most frequently in the body of the pancreas (49 (48.0%)), followed by the head (36 (35.3%)) and tail (17 (16.7%)). The sizes of the PCLs ranged from 5 mm to 75 mm.

The success rates for delineating PCLs using US are shown in Table 2. The mean size difference of the lesions between US and EUS was 1.5 mm (range, −15 mm to +10 mm). The ICC of US with the corresponding EUS size of the PCLs showed very good reliability, with a value of 0.978 (*p* < 0.001).

The κ value (strength of agreement) between US and EUS for the locularity and size of individual compartments of the PCLs was 0.529, indicating moderate agreement (Table 3). The concordance rate between US and EUS for the locularity and size of individual compartments of PCLs was the highest for unilocular PCLs at 96.8% (30/31), followed by multilocular microcystic PCLs (77.8%, 7/9).

The κ value was calculated for other morphological characteristics, including worrisome features (Table 4). Among the worrisome features, the κ value for pancreatic duct dilation was 0.882 (*p* < 0.001), showing good agreement, whereas the κ values for the solid component and cystic wall/septal thickening were 0.481 (*p* < 0.001) and 0.395 (*p* < 0.001), respectively, which indicated moderate agreement. As for other morphologic characteristics, the κ values for a lobulated margin, smooth margin with internal septation, and multifocality were superior to those of other characteristics, showing good agreements of 0.637 (*p* < 0.001), 0.650 (*p* < 0.001), and 0.633 (*p* < 0.001), respectively.

## 4. Discussion

With advances in imaging technology, PCLs are frequently revealed by abdominal US or transverse section imaging examinations. Despite the potential benefits of US in PCL follow-up assessments, its application has not been fully evaluated in previous studies. The sensitivity of US for detecting PCLs has been reported to range from 70.2% to 88.3% [7]. The relatively low sensitivity of US for PCL detection can be influenced by various confounding factors, such as the disruption of ultrasound transmission related to the presence of bowel gas or obesity, retroperitoneal location of the pancreas, and high operator dependency [7]. Moreover, there is little evidence regarding the efficacy of US for the detection and monitoring of the significant worrisome features of PCLs.

In clinical practice, it is necessary to determine the usefulness of US, especially for the follow-up of PCLs, since US can be used to overcome the practical constraints of other standard imaging modalities. These constraints include patients who are not suitable for sedation, those with decreased renal function and therefore cannot be administered contrast agent, or those who do not have the financial means to pay for more advanced medical procedures.

However, among various international guidelines, only the Italian guidelines issued in 2014 recommended US as a follow-up imaging tool for PCLs. In that guideline, US imaging was recommended for the follow-up of PCLs until there is a change in size [13]. According to a retrospective study by Jeon et al., which included 1064 PCLs, the sensitivity of US for PCL detection was 88.3% [14]. In that study, the US detection rate of PCLs was significantly improved from 49.2% to 86.7% with other correlative imaging techniques such as MRI, CT, and EUS. Hence, they concluded that US is a useful tool for the follow-up of PCLs that are incidentally detected through CT, MRI, or EUS [14].

In US, the use of several different scan planes can facilitate a better delineation and characterization of PCLs by rotation, tilting, and movement of the US probe according to the intention of the operator, as the US probe is located outside the body. In contrast, in EUS, the selection of the scan plane is more limited, as the ultrasound transducer is located inside the body. Nevertheless, EUS image quality is usually superior to that of US images, as EUS allows for visualization in closer proximity to the pancreas, whereas the quality of US images is strongly affected by the presence of duodenal gas or subcutaneous fat tissue. Therefore, during US, various scan planes should be utilized to obtain the best images of PCLs. In the current study, the reliability between US and EUS for measuring the size of the PCLs was very good, with an ICC value of 0.978. PCLs of the body portion were well detected by US, with a delineation success rate of 91.8%, whereas the delineation success rates for head and tail lesions were not satisfactory at 77.8% and 70.6%, respectively. Limitations related to a poor ultrasonic window associated with penetration depth can prohibit the successful delineation of PCLs located in the head or tail. Moreover, microcystic lesions may not be discriminated from the coarse pancreatic parenchyma in patients with chronic pancreatitis. Therefore, US may be a reasonable option for follow-up assessments of PCL size, as long as the lesion is delineable on US. Moreover, in the current study, the sensitivity of US for discriminating unilocular cysts as a reference standard using EUS was 96.8%, whereas the sensitivity for multilocular cysts was not satisfactory. Hence, unilocular cysts confirmed by EUS may be cautiously followed up using US.

According to the revised Fukuoka guideline of 2017, morphological characteristics such as main pancreatic duct dilation, mural nodules, and wall thickening are important for the diagnosis and prediction of malignant transformation of PCLs [15]. Therefore, the most important issue in PCL follow-up is the timely detection of newly developed, high-risk stigmata or worrisome features of PCLs. In a large-scale study by Ikeda et al., US was shown to be capable of detecting most pancreatic duct dilations larger than 3 mm [16]. However, there has been limited evaluation of the role of US in detecting other worrisome features in previous studies [16]. In our study, US showed excellent agreement with the corresponding EUS findings for detecting main pancreatic duct dilation. In contrast, the results related to cyst walls and multifocal cysts showed low degrees of agreement between US and EUS. Therefore, even when US is used for the follow-up of PCLs, standard imaging such as CT, MRI, or EUS is alternatively necessary, as US presents false-negative findings for those worrisome features. In the current study, a smooth margin with internal septation, most likely suggesting a mucinous cystic neoplasm, showed a relatively good agreement between US and EUS, as compared with other morphologic characteristics not included in the worrisome features. However, further data accumulation is needed before this result can be clinically applicable. 

During the follow-up of PCLs, it is important to monitor the development of metachronous pancreatic cancer in a location that differs from that of the original mucinous cyst [17]. However, there are limitations to the use of US in the surveillance of small metachronous cancer lesions as compared with standard imaging modalities. Hence, if US is adopted as a follow-up tool for monitoring PCLs, CT, MR or EUS should be performed between US procedures to compensate for the relatively low spatial resolution of US for the delineation of the pancreas.

Although our study is the first to evaluate the capability of US for the morphological characterization of PCL as a reference standard using EUS, there are some limitations to consider. First, our study included a small population from a single tertiary center; therefore, the measured outcomes may not be generalized to the entire population. Second, our study was limited by a potential bias related to its retrospective nature.

In conclusion, US may be useful for monitoring the growth of PCLs, especially unilocular PCLs, and changes in pancreatic duct dilation during follow-up. However, US shows limited applicability in the diagnosis and monitoring of mural nodules and the wall thickness of PCLs. Further large-scale, multicenter studies are needed to verify the usefulness of US for the evaluation and follow-up of PCLs.

## Figures and Tables

**Table 1:** Summary of patient demographics.

	Number of PCLs/Number of Patients (102/92)
Age, years	61.9 ± 12.7
Sex (M/F)	41/51
History of pancreatitis	10 (9.8%)
History of alcohol abuse	11 (10.8%)
Body weight, kg	61.1 ± 11.0
BMI, kg/m^2^	23.1 ± 3.5
Initial diagnostic modality (CT/US)	61 (59.8%)/41 (40.2%)

Data are presented as the mean ± SD or numbers (percentages). PCL, pancreatic cystic lesion; BMI, body mass index; CT, computed tomography; US, ultrasonography; EUS, endoscopic ultrasonography.

**Table 2:** Capability of US to delineate PCLs as a reference standard using EUS, and size differences of the lesions between the two modalities.

	US/EUS (%)
Delineation of cyst	85/102 (83.3)
Head	−28/36 (77.8)
Body	45/49 (91.8)
Tail	12/17 (70.6)
Size of the lesions based on EUS, mm	
≤10 mm	18 (17.6%)
>10 mm ≤20 mm	43 (42.2%)
>20 mm ≤30 mm	18 (17.6%)
>30 mm	23 (22.5%)
Difference in cyst size between US and EUS	1.5 mm (−15–+10), ICC *: 0.978 (*p* < 0.001)

US, ultrasonography; PCL, pancreatic cystic lesion; EUS, endoscopic ultrasonography; ICC, intraclass correlation coefficient; * Intraclass correlation coefficient value (strength of reliability): >0.80, very good; 0.60–0.79, moderate; <0.60, not reliable.

**Table 3:** Capability of US to characterize the locularity and the size of individual compartments of PCLs as a reference standard using EUS.

	US/EUS (%)	
Morphologic characteristics	59/85 (69.4)	κ value:0.529 *
Unilocular	30/31 (96.8)	
Multilocular microcystic	7/9 (77.8)	
Multilocular macrocystic	20/38 (52.6)	
Multilocular micro and macrocystic	2/7 (28.6)	

US, ultrasonography; PCL, pancreatic cystic lesion; EUS, endoscopic ultrasonography. * κ value (strength of agreement): 0.81–1.00, excellent; 0.61–0.81, good; 0.41–0.60, moderate; 0,21–0.40, fair; 0–0.20, poor.

**Table 4:** Capability of US to characterize PCLs as a reference standard using EUS.

	Sensitivity (%)	κ Value
MPD dilation *	14/17 (82.4)	0.882
Cyst wall or septal thickening **	3/11 (27.3)	0.395
Solid component	5/14 (35.7)	0.481
Suspicious communication with MPD	10/32 (31.3)	0.167
Lobulated margin	12/21 (57.1)	0.637
Honeycomb appearance	6/12 (50.0)	0.548
Smooth margin with septation	3/6 (50.0)	0.650
Multifocal	10/19 (52.6)	0.633
Pleomorphic cystic	5/11 (45.5)	0.592
Grape-like	6/13 (46.2)	0.592
Clubbed finger-like	9/19 (47.4)	0.583

US, ultrasonography; PCL, pancreatic cystic lesion; EUS, endoscopic ultrasonography; MPD, main pancreatic duct. * head ≥ 5 mm or body/tail ≥ 4 mm; ** >3 mm.

## Data Availability

Data are available upon reasonable request.

## References

[1] Mohamed E., Jackson R., Halloran C.M., Ghaneh P. (2018). Role of Radiological Imaging in the Diagnosis and Characterization of Pancreatic Cystic Lesions: A Systematic Review. Pancreas.

[2] Sun L., Wang Y., Jiang F., Qian W., Shao C., Jin Z. (2019). Prevalence of pancreatic cystic lesions detected by magnetic resonance imaging in the Chinese population. J. Gastroenterol. Hepatol..

[3] Choi J.H., Lee S.H. (2019). Endoscopic Ultrasound-based Approach in the Diagnosis and Treatment for Pancreatic Cystic Lesions. Korean J. Pancreas Biliary Tract.

[4] Lee Y.S., Paik K.H., Kim H.W., Lee J.C., Kim J., Hwang J.H. (2015). Comparison of Endoscopic Ultrasonography, Computed Tomography, and Magnetic Resonance Imaging for Pancreas Cystic Lesions. Medicine (Baltimore).

[5] Choi S.H., Park S.H., Kim K.W., Lee J.Y., Lee S.S. (2017). Progression of Unresected Intraductal Papillary Mucinous Neoplasms of the Pancreas to Cancer: A Systematic Review and Meta-analysis. Clin. Gastroenterol. Hepatol..

[6] Ohno E., Hirooka Y., Kawashima H., Ishikawa T., Fujishiro M. (2020). Endoscopic ultrasonography for the evaluation of pancreatic cystic neoplasms. J. Med. Ultrason. (2001).

[7] Hashimoto S., Hirooka Y., Kawabe N., Nakaoka K., Yoshioka K. (2020). Role of transabdominal ultrasonography in the diagnosis of pancreatic cystic lesions. J. Med. Ultrason. (2001).

[8] Oh H.C., Kim M.H., Hwang C.Y., Lee T.Y., Lee S.S., Seo D.W., Lee S.K. (2008). Cystic lesions of the pancreas: Challenging issues in clinical practice. Am. J. Gastroenterol..

[9] Sahani D.V., Kadavigere R., Saokar A., Fernandez-del Castillo C., Brugge W.R., Hahn P.F. (2005). Cystic pancreatic lesions: A simple imaging-based classification system for guiding management. Radiographics.

[10] Fan Z., Yan K., Wang Y., Qiu J., Wu W., Yang L., Chen M. (2015). Application of Contrast-Enhanced Ultrasound in Cystic Pancreatic Lesions Using a Simplified Classification Diagnostic Criterion. Biomed. Res. Int..

[11] Koo T.K., Li M.Y. (2016). A Guideline of Selecting and Reporting Intraclass Correlation Coefficients for Reliability Research. J. Chiropr. Med..

[12] Landis J.R., Koch G.G. (1977). The measurement of observer agreement for categorical data. Biometrics.

[13] Buscarini E., Pezzilli R., Cannizzaro R., De Angelis C., Gion M., Morana G., Zamboni G., Arcidiacono P., Italian Association of Hospital Gastroenterologists and Endoscopists, Italian Association for the Study of the Pancreas (2014). Italian consensus guidelines for the diagnostic work-up and follow-up of cystic pancreatic neoplasms. Dig. Liver Dis..

[14] Jeon J.H., Kim J.H., Joo I., Lee S., Choi S.Y., Han J.K. (2018). Transabdominal Ultrasound Detection of Pancreatic Cysts Incidentally Detected at CT, MRI, or Endoscopic Ultrasound. AJR Am. J. Roentgenol..

[15] Tanaka M., Fernandez-Del Castillo C., Kamisawa T., Jang J.Y., Levy P., Ohtsuka T., Salvia R., Shimizu Y., Tada M., Wolfgang C.L. (2017). Revisions of international consensus Fukuoka guidelines for the management of IPMN of the pancreas. Pancreatology.

[16] Ikeda M., Sato T., Morozumi A., Fujino M.A., Yoda Y., Ochiai M., Kobayashi K. (1994). Morphologic changes in the pancreas detected by screening ultrasonography in a mass survey, with special reference to main duct dilatation, cyst formation, and calcification. Pancreas.

[17] Yamaguchi K., Ohuchida J., Ohtsuka T., Nakano K., Tanaka M. (2002). Intraductal papillary-mucinous tumor of the pancreas concomitant with ductal carcinoma of the pancreas. Pancreatology.

